# Nickel-Glycerolate/MOF-Based Electrode Materials for Energy Storage and Water-Splitting Applications

**DOI:** 10.1021/acsomega.6c06663

**Published:** 2026-09-23

**Authors:** Iara Schlag Durães Campos, Abelardo Gregório Banze, Tarso Leandro Bastos, Raylander Rodrigues Silva, Niélcio Batista da Silva Filho, Leonardo Morais Da Silva, Josué Martins Gonçalves, Paulo Roberto Martins

**Affiliations:** † Chemistry Institute, Federal University of Goias, Av. Esperança S/N, Goiânia, GO 74690-900, Brazil; ‡ Center of Excellence in Hydrogen and Sustainable Energy Technologies. Chemistry Institute, Federal University of Goias, Av. Esperança S/N, Goiânia, GO 74690-900, Brazil; § Department of Chemistry, Laboratory of Fundamental and Applied Electrochemistry, Federal University of Jequitinhonha and Mucuri’s Valley, Rodovia MGT 367, Km 583, 5000, Alto da Jacuba, Diamantina, MG 39100-000, Brazil; ∥ Mackenzie Institute for Research in Graphene and Nanotechnologies (MackGraphe), Mackenzie Presbyterian Institute, Consolação Street 930, São Paulo, São Paulo 01302-907, Brazil; ⊥ Mackenzie School of Engineering, Mackenzie Presbyterian University, Consolação Street 930, São Paulo, São Paulo 01302-907, Brazil

## Abstract

Herein, binary coordination compounds based on Ni-Gly/ZIF-67 and Ni-Gly/CoFe-ZIF-67 were employed as electrode materials for energy storage and water-splitting applications, respectively. The structure and morphology of the binary electrodes were characterized by XRD, XPS, and SEM. Electrochemical studies (cyclic voltammetry and electrochemical impedance spectroscopy) revealed that the binary materials exhibited distinct electrochemical behavior for energy storage and water splitting. In fact, whereas Ni-Gly/ZIF-67 was used as an electrode material for energy storage, Ni-Gly/CoFe-ZIF-67 was used as an electrocatalyst for water splitting. The Ni-Gly/ZIF-67 electrode material achieved a high specific capacity of 106.9 mA h g^–1^ at 1.0 A g^–1^ and retained 71.6% of its capacity after 5000 cycles, combining pseudocapacitive/battery-type behavior. Furthermore, a symmetric supercapacitor device was assembled, achieving maximum energy and power densities of 0.765 W h kg^–1^ and 6076 W kg^–1^, respectively. The Ni-Gly/CoFe-ZIF-67 electrode material exhibited excellent oxygen evolution reaction (OER) activity, with an overpotential of 430 mV at 10 mA cm^–2^, a Tafel slope of 108 mV dec^–1^, and stable operation for 10 h. These results demonstrate a synergism between MOF- and Ni-Gly-derived frameworks, providing valuable insights for the rational design of sustainable multifunctional electrodes for integrated energy storage and conversion systems.

## Graphical abstract

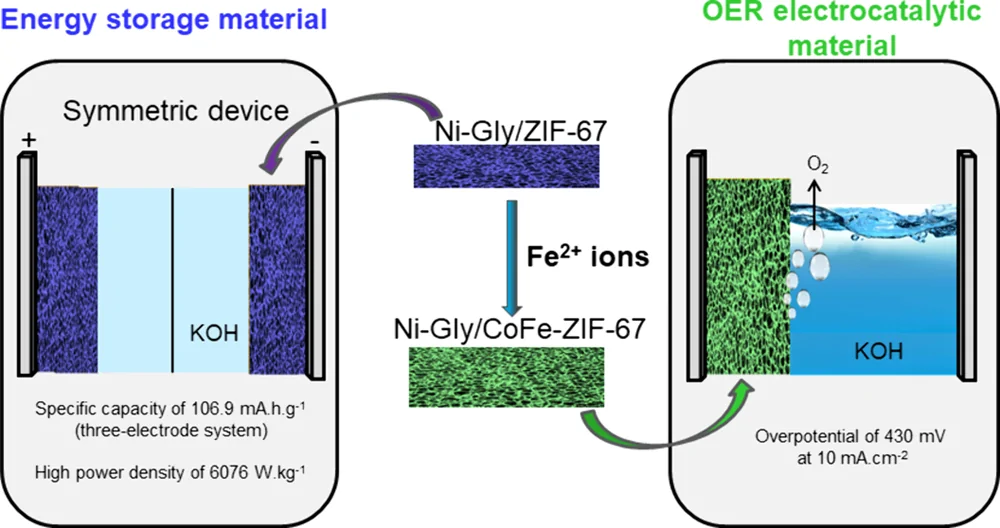

## 1 Introduction

The transition to sustainable and efficient energy systems requires the development of energy materials capable of operating in multifunctional applications.^1^ Among the most pressing global challenges are large-scale electrical energy storage and the production of clean fuels via renewable routes.^2^ Even though these technologies are intended for different energy demands, both require electrode materials with abundant electroactive sites, efficient charge transport, and long-term stability. Consequently, electrode material platforms whose electrochemical properties can be rationally tailored for distinct energy storage and conversion applications are of particular interest.

Regarding energy storage, supercapacitors and battery-type systems are recognized as complementary technologies, the former offer high-density power and long cycle life, while the latter exhibit high-density energy.^1,2^ Additionally, the performance of these devices depends heavily on the electrode material, particularly its porosity, surface area, and conductivity.^3^ On the other hand, water splitting has received significant attention as a sustainable approach for producing the so-called green hydrogen.^4^ This process produces hydrogen and oxygen gases as clean products, with a theoretical potential of 1.23 V. It involves two half-reactions, where the oxygen evolution reaction (OER) occurs at the anode, and the hydrogen evolution reaction (HER) occurs at the cathode. Due to slow kinetics, particularly for OER, substantial overpotentials are required for efficient operation.^5,6^ Recent research focuses on the search for efficient, robust, and low-cost electrocatalysts, since traditional noble-metal catalysts such as platinum (for HER) and iridium or ruthenium oxides (for OER) are expensive and scarce.^5–7^ Consequently, compositional tuning of the same coordination compound platform to satisfy the distinct requirements of electrochemical energy storage and energy conversion has emerged as a crucial area of research.

In this context, the so-called *coordination compounds* have been extensively reported as an important class of materials with great prospects for energy conversion and storage. Among the most important coordination compounds, metal–organic frameworks (MOFs) and their derivatives are promising platforms due to their large surface areas, tunable porosity, and chemical versatility.^6,8^ Notably, the cobalt-based zeolitic imidazolate framework (ZIF-67) has been widely investigated. Studies by Wen et al., Yang et al., and Catterjee et al. have revealed that introducing a second transition metal, such as iron (Fe), into the ZIF-67 structure can create synergistic electronic interactions, significantly enhancing the OER activity by optimizing the adsorption energies of oxygen-containing intermediates.^6,8,9^ These findings demonstrate that rational compositional modification of MOFs represents an effective strategy for tailoring their electrochemical properties toward specific applications.

Beyond classical MOFs, metal glycerolates (M-Gly) and their derivatives have emerged as a promising class of electrode materials, spanning a wide compositional space from metal oxides and hydroxides to metal sulfides and phosphides.^10^ These materials have shown great potential as electrode materials (or scaffolds and precursors) for supercapacitors and batteries, as well as for electrocatalysis in water splitting. In fact, recent studies have reported promising results for the use of M-Gly as precursors for the oxygen evolution reaction (OER),^11^ as the *in situ* formation of metal oxyhydroxide (MOOH) species, during electrochemical process, on the electrode surface is key to achieving high-performance catalysts. The superior activity is attributed to the abundant MOOH active sites generated by electrochemical activation in an alkaline electrolyte commonly used in OER conditions.^12^ Recent insights from Gonçalves et al.^13^ challenge traditional synthesis pathways, revealing that materials long thought to be metal hydroxides (e.g., those synthesized via the Tower’s method) are, in fact, metal-glycerolates (Ni-Gly). These layered metal–organic precursors, upon controlled reaction with aqueous alkali, can be transformed into highly active nickel oxyhydroxide (NiOOH) phases for electrocatalysis and energy storage, thereby opening a new avenue for precursor design.^14,15^ This underscores the continuous evolution in material synthesis, where understanding the true nature of precursors is key to engineering advanced electrodes with dual electrochemical functionalities for both energy storage and conversion.

Furthermore, the strategic coupling of MOFs with battery-type metal hydroxides, such as Ni­(OH)_2_, is a powerful design approach to create multifunctional electrodes. By combining the porous architecture of MOFs with the high Faradaic activity of nickel-based compounds, these hybrid systems seek to overcome the intrinsic limitations of the individual components while taking advantage of their complementary electrochemical properties. This approach, as explored in recent works, leverages the high conductivity and rich redox chemistry of the hierarchical porous scaffold while integrating the high charge-storage properties of the metal hydroxide.^14^ A notable strategy demonstrated by Li et al.^15^ involved the synthesis of Ni­(OH)_2_@Co/C nanocomposites with a core–shell architecture, in which ZIF-67-derived Co/C served as a conductive core and ultrathin Ni­(OH)_2_ nanosheets formed the active shell. The resulting mixed electrode delivered an impressive specific capacitance of 952 F g^–1^ at 0.5 A g^–1^ and maintained 84% of its capacity after 10,000 cycles, effectively addressing the intrinsic conductivity and cycling stability limitations of pristine Ni­(OH)_2_. These studies clearly demonstrate the benefits of integrating coordination frameworks with nickel-based electroactive materials. However, they have predominantly focused on conventional nickel hydroxides, whereas the electrochemical behavior of nickel glycerolates coupled with MOFs remains considerably less explored. Thus, nickel glycerolates possess a distinct coordination environment that may facilitate integration with ZIF-based frameworks, and active NiOOH is electrochemically formed in situ. However, integrating nickel glycerolates directly with monometallic and bimetallic ZIF frameworks to tailor targeted electrochemical functions remains underexplored.

Herein, we investigate two binary electrodes with distinct application targets, Ni-Gly/ZIF-67 designed specifically for energy storage, and Ni-Gly/CoFe-ZIF-67 tailored for the oxygen evolution reaction (OER). This work provides a systematic understanding of how structural characteristics and chemical composition govern the electrochemical properties and specific performance of these advanced electrode materials.

## 2 Experimental Section

### 2.1 Synthesis

ZIF-67 was synthesized following a previously reported procedure.^16^ Initially, Co­(C_2_H_3_O)_2_·6H_2_O (0.312 g) was added to methanol (25 mL), and the mixture was allowed to rest for 10 min. Subsequently, a solution of 2-methylimidazole (0.410 g) in methanol was added to the mixture, which was then stirred under magnetic conditions for 12 h. Finally, a purple colloidal solution of ZIF-67 was obtained. CoFe-ZIF-67 was synthesized by mixing Co­(C_2_H_3_O)_2_·6H_2_O (0.233 g) and FeSO_4_·7H_2_O (0.087 g) in methanol (25 mL). After the static period, 2-methylimidazole (0.410 g) in methanol was added to the reaction medium, and the mixture was stirred magnetically for 12 h. At the end of the reaction, a purple colloidal solution of CoFe-ZIF-67 was obtained. In order to obtain Ni-Gly, Ni­(C_2_H_3_O)_2_·4H_2_O (1.394 g) was dissolved in 15 mL of glycerol (solution A), followed by the addition of a KOH solution (0.628 g in 10 mL of *n*-butanol) into solution A, as reported previously.^13^ The resulting mixture was magnetically stirred for 6 h, yielding a green colloidal solution.

### 2.2 Characterization

The samples were characterized through X-ray diffraction (Shimadzu XRD-6000 diffractometer with Cu Kα radiation of 1.5418 Å). Scanning Electron Microscopy (SEM-FEG Jeol JSM7100F) with an accelerating voltage of 2.5 kV, and X-ray photoelectron spectroscopy (XPS - Thermo Fisher Scientific, UK), where the analyses were carried out for Ni-Gly/ZIF-67 and Ni-Gly/CoFe-ZIF-67 using Al as the excitation source. In addition, an FTIR spectrophotometer (PerkinElmer Spectrum 400) was used to record spectra of ZIF-67 and CoFe-ZIF-67 in the region of 400 – 4000 cm^–1^.

### 2.3 Preparation of Electrodes and Electrochemical Measurements

The samples were deposited onto a Fluorine-doped Tin Oxide (FTO) glass substrate. Prior to deposition, the FTO was washed with soap and rinsed copiously with water. An area of 1.0 cm^2^ was defined using adhesive tape, followed by the drop-casting of 10 μL of either ZIF-67 or CoFe-ZIF-67 colloidal solution. The substrates were then heated at 80 °C for 2 h in a conventional oven. After cooling, 10 μL of Ni-Gly colloidal solution was deposited onto the modified FTO and spin-coated at 2500 rpm for 1 min to ensure film homogeneity. Finally, the substrates were thermally treated at 240 °C for 30 min to remove residual solvents from the synthesis, yielding Ni-Gly/ZIF-67 and Ni-Gly/CoFe-ZIF-67. For comparison, ZIF-67, CoFe-ZIF-67, and Ni-Gly films were also deposited on FTO glass substrates.

All electrochemical experiments were performed using a Potentiostat/Galvanostat from AUTOLAB (AUT84605) and a three-electrode cell configuration with an FTO glass substrate modified as the working electrode, an Ag/AgCl (KCl_–saturated_) reference electrode, and a platinum wire as the auxiliary electrode. The supporting electrolyte is a KOH (1.0 mol L^–1^) solution. The electrochemical behavior of the samples (ZIF-67, CoFe-ZIF-67, Ni-Gly, Ni-Gly/ZIF-67_,_ and Ni-Gly/CoFe-ZIF-67) was evaluated by Cyclic Voltammetry (CV) and Electrochemical Impedance Spectroscopy (EIS). CVs were recorded at 20 mV s^–1^ in the – 0.1 to 0.55 V potential range, and EIS was carried out applying a potential of 0.50 V using the frequency range of 0.01 Hz to 100 kHz with a sinusoidal AC perturbation with an amplitude of 10 mV at 25 °C. The galvanostatic charge–discharge (GCD) curves were performed within a potential window of 0 to 0.43 V using different current densities (e.g., 1 to 12 A g^–1^). Linear sweep voltammetry (LSV) was recorded in the – 0.10 to 0.55 V range at a scan rate of 5 mV s^–1^, and Chronopotentiometric measurements were conducted at a constant current of 10 mA for a continuous period of 10 h.

The specific capacity referring to the *battery-like* electrode materials was determined using eq 1

1 $$C_{s}^{*}=\frac{I\Delta t}{m}$$

where *C*_s_* is the specific capacity (A s kg^–1^), *I* is the applied current (A), *t* is the time (s), and *m* is the active mass (kg) of the electrode material.

A symmetrical Ni-Gly/ZIF-67//Ni-Gly/ZIF-67 device was assembled as follows: the electrode slurry was prepared using activated carbon, PVDF, and Black Pearls in a mass ratio of 4:1:1, dispersed in 3 mL of *N*-methyl-2-pyrrolidone (NMP). Subsequently, 0.6 mL of ZIF-67 suspension and 0.6 mL of Ni-Gly suspension were added to the mixture. The slurry was stirred for 24 h and then coated onto a copper foil current collector (16 mm diameter). The total mass loading of the electrodes was 1.59 mg cm^–2^. Subsequently, the electrodes were assembled into a CR2032 coin cell, using a cellulosic filter paper as the separator and 1.0 mol L^–1^ KOH as the electrolyte. The electrochemical performance of the symmetrical Ni-Gly/ZIF-67//Ni-Gly/ZIF-67 device was determined through GCD curves (0.2 to 0.8 V) at 1 mA g^–1^.

The Ragone plot for supercapacitor materials was obtained from GCD data using eqs 2 and 3

2 $$E=\frac{C_{s}{\left(\Delta U\right)}^{2}}{2m}$$

3 $$P=\frac{3600E}{\Delta t}$$

where *E* is the energy density (J kg^–1^), *C*_*s*_ is the specific capacitance (F kg^–1^), Δ*U* is the voltage/potential window, *m* is the electrode active mass (g), and *P* is the specific power (W h g^–1^).

In the case of battery-like materials, the stored energy (*E*_Bat_) was determined as follows

4 $$E_{Bat}=I\Delta Ut$$

where *I* is the constant applied current (A), and *t* is the time (s) in GCD experiments.

All specific energy and power values mentioned above were double-checked using the approach proposed recently by Da Silva et al.,^17^ where the average energy and power values are calculated from numeric integration of the experimental GCD plots as follows:

5 $${\left\langle E\right\rangle }_{SCs}=\frac{I\int_{0}^{t}\Delta U\left(t\right)dt}{2}$$

6 $${\left\langle E\right\rangle }_{Bat}=I\int_{0}^{t}\Delta U\left(t\right)dt$$

7 $${\left\langle P\right\rangle }_{SCs/Bat}=\frac{{\left\langle E\right\rangle }_{SCs/Bat}}{\Delta t}$$

As with the other electrochemical tests, the OER catalytic activity of the electrodes modified with CoFe-ZIF-67, Ni-Gly, and Ni-Gly/CoFe-ZIF-67 was evaluated using the same electrochemical setup. Initially, the electrodes were subjected to 40 CV cycles to stabilize the samples in the electrolyte and promote the activation of nickel glycerolate-based precursors, leading to the *in situ* formation of NiOOH, a recognized active catalyst for water splitting. Subsequently, LSV results were converted to the RHE scale to facilitate data comparison.

The long-term electrochemical stability of the binary electrode was assessed by chronopotentiometry. Additionally, Tafel plots were obtained to analyze electrochemical reaction mechanisms and kinetics.

## 3 Results and Discussion

The FTIR analyses of ZIF-67 and CoFe-ZIF-67 were carried out, and the spectra are presented in Figure 1A. It was observed that the band at 425 cm^–1^, common for both samples, is assigned to the metal–nitrogen (M-N) stretching vibration, indicating coordination between the imidazolate ligand and the metal centers.^18^ Comparison of the two spectra further reveals that the characteristic bands located at 1575 cm^–1^, corresponding to the C=N stretching vibration of the imidazole ring, 1420 cm^–1^, attributed to in-plane deformation of the imidazole ring, 1120 cm^–1^, assigned to C–N stretching, 750 cm^–1^, and 677 cm^–1^, associated with out-of-plane bending vibrations, remain essentially unchanged in their vibrational frequencies. Additional evidence suggesting the possible incorporation of iron is provided by the appearance of a distinct absorption band centered at approximately 620 cm^–1^, which is absent in the spectrum of pristine ZIF-67. The appearance of this new band indicates a modification of the coordination environment and may be associated with changes arising from the presence of iron species in the bimetallic framework. In addition, the C–H stretching vibrations (2900 – 3100 cm^–1^) were observed for both samples.^19^

**Figure 1:**
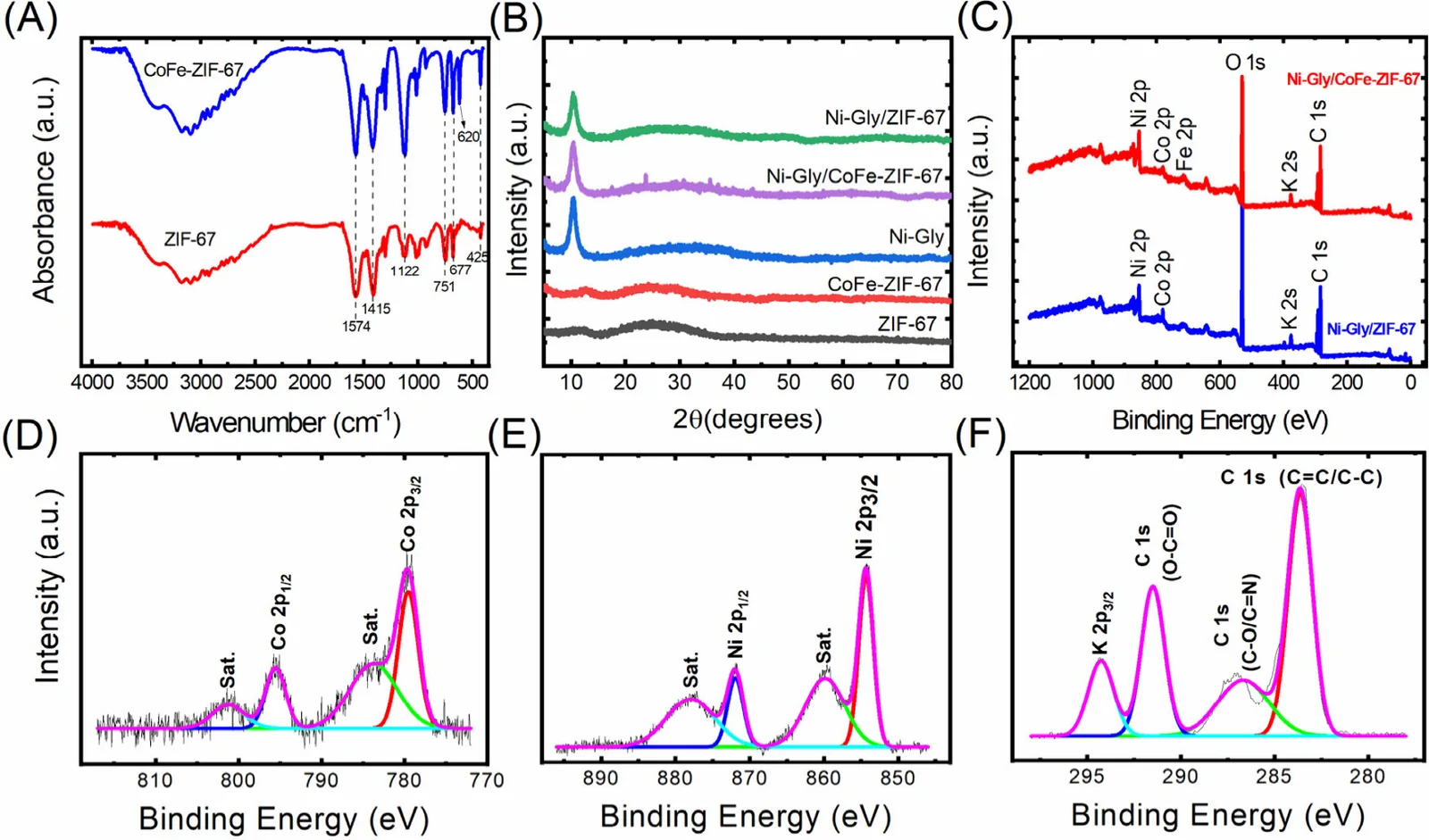
Different ex-situ findings. (A) FTIR spectra of ZIF-67 and CoFe-ZIF-67. (B) XRD patterns for the samples. (C) Survey XPS spectrum of Ni-Gly/ZIF-67 and Ni-Gly/CoFe-ZIF-67. High-resolution XPS spectra of Ni-Gly/ZIF-67: (D) Co 2p, (E) Ni 2p, and (F) C 1s.

The XRD patterns of Ni-Gly, ZIF-67, CoFe-ZIF-67, Ni-Gly/ZIF-67, and Ni-Gly/CoFe-ZIF-67 based electrodes are shown in Figure 1B. ZIF-67 and CoFe-ZIF-67 displayed identical patterns, with no crystalline diffraction peaks, consistent with previous reports.^16^ Ni-Gly exhibited a single diffraction peak at 2θ = 10.26°, a typical structural feature of metal–glycerolates, composed of layered metal–oxygen frameworks interspaced with coordinated glycerolate ions.^10^ As reported by Gonçalves et al.,^13^ this material is a mixture of Ni^2+^ complexes coordinated to O-donor ligands (e.g., glycerol, glycerolate, acetate, OH^–^), which converts to layered Ni-Gly upon heat treatment. Notably, Ni-Gly/ZIF-67 and Ni-Gly/CoFe-ZIF-67 also show a single diffraction peak in the same 2θ region as Ni-Gly, as expected for a homogeneous film obtained by spin-coating.

XPS analyses were conducted on Ni-Gly/ZIF-67 and Ni-Gly/CoFe-ZIF-67 to determine their chemical composition and the oxidation states of the constituent elements. The survey spectra (Figure 1C) showed characteristic peaks corresponding to Co, Ni, Fe, C, O, and residual K, as byproducts of the Ni-Gly synthesis.

High-resolution spectra for the Ni-Gly/ZIF-67 electrode material (Figures 1D–1F) reveal well-defined features for each element. The Co 2p spectrum (Figure 1D) exhibits main peaks at 779.65 and 795.55 eV, with satellites at 783.75 and 801.23 eV, which are assigned to Co^2+^/Co^3+^ ions.^20^ Similarly, the Ni 2p spectrum (Figure 1E) exhibits peaks at 854.34 and 872.03 eV, along with their corresponding satellites at 859.85 and 877.98 eV, consistent with Ni^2+^/Ni^3+^ ions. Finally, the C 1s spectrum (Figure 1F) was deconvoluted into four peaks. The signals at 283.62, 286.67, and 291.49 eV are assigned to C=C/C–C, C–O/C=N, and O–C=O bonds, respectively, while the peak at 294.26 eV is associated with K^+^ from the use of KOH in the synthesis.^13,16^ Ni-Gly/CoFe-ZIF-67 (Figure S1), the Co 2p spectrum displayed main peaks at 779.70 and 795.60 eV, with satellites at 784.15 and 801.64 eV, related to Co^2+^/Co^3+^ ions. In the case of Ni 2p, peaks were observed at 854.51 and 872.14 eV, with satellites at 860.24 and 878.31 eV. For C 1s, the signals at 283.80, 286.50, and 291.73 eV are assigned to C=C/C–C, C–O/C=N, and O–C=O bonds, respectively. The presence of mixed-valence iron in the modified framework was confirmed in the fitted high-resolution Fe 2p spectrum. In fact, the Fe 2p spectrum presented peaks related to Fe^2+^ species at 712.20 and 722.75 eV, in addition to a peak at 732.40 eV correlated with the Fe^3+^ ion.^21^ When compared with Ni-Gly/ZIF-67, the Ni-Gly/CoFe-ZIF-67 exhibited a slight positive shift for Ni 2p, particularly the Ni 2p 3/2 peak (∼0.17 eV), while Co 2p peaks remain nearly unchanged. This small positive shift indicates a subtle decrease in the electron density around Ni, suggesting that the introduction of Fe into ZIF-67 induces an electronic interaction with the Ni-Gly matrix. Such electronic modulation may facilitate the electrochemical oxidation of Ni^2+^ into electrocatalytically active NiOOH species under alkaline conditions, thereby contributing to the enhanced OER performance.^22^

Figure 2 shows SEM images and elemental mapping for Ni-Gly/ZIF-67 and Ni-Gly/CoFe-ZIF-67. As shown in Figure 2A, the Ni-Gly/ZIF-67 sample exhibits several cracks, which are likely due to the heating process during sample preparation. Furthermore, the porous nature of the Ni-Gly/ZIF-67-based film is clearly visible in Figure 2B. Neither the cracks nor the porous structure seems to originate from ZIF-67 and CoFe-ZIF-67 samples, since the SEM images of ZIF-67 and CoFe-ZIF-67 samples (Figure S2A–S2F) reveal a uniform distribution of the material on the substrate, forming a continuous thin film without signs of agglomeration, cracks, or porosity. Therefore, this morphology seems to be characteristic of the Ni-Gly-based sample. In fact, both cracks and porous structure are observed on the pristine Ni-Gly electrode, as evidenced in Figure S2G-S2I [16,23,24]. The Energy Dispersive Spectroscopy (EDS) technique revealed that the elements N, Ni, Co, C, and O are homogeneously distributed throughout the sample, as shown in Figure 2C. SEM images of the Ni-Gly/CoFe-ZIF-67 sample (Figure 2D-2F) display the same morphology as that of Ni-Gly/ZIF-67. In addition to Ni, Co, C, N, and O elements, the presence of Fe in the sample was also confirmed.

**Figure 2:**
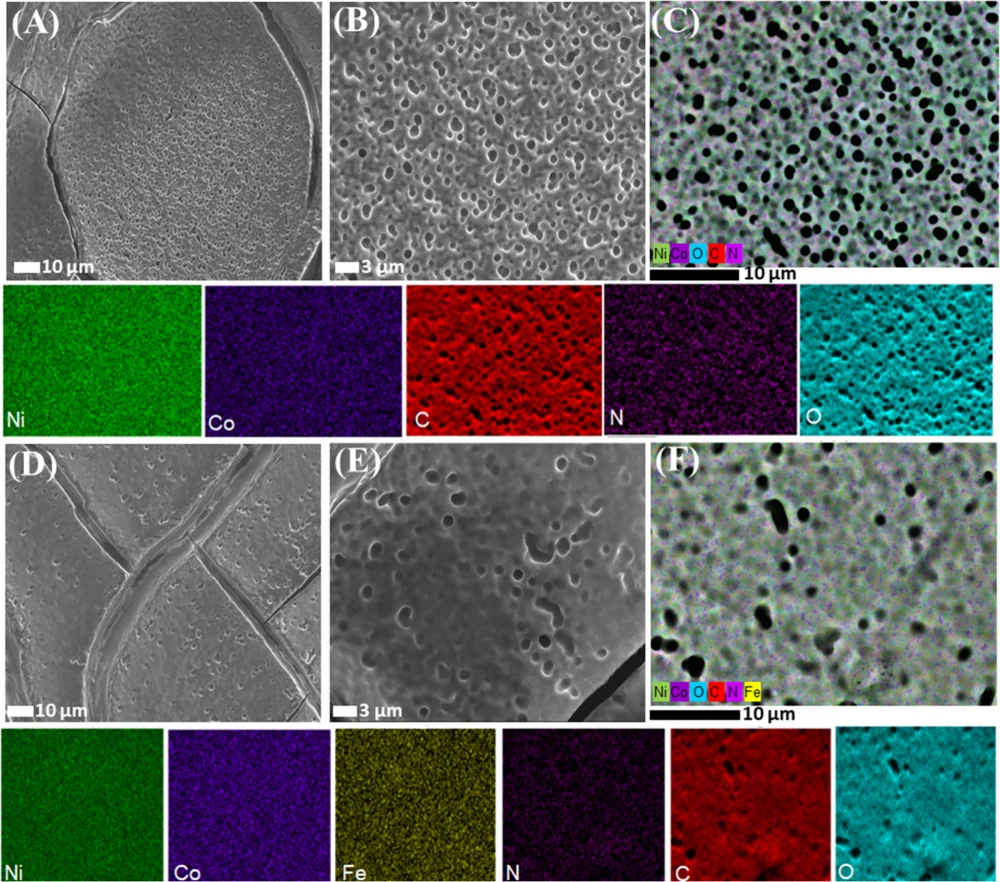
SEM images (A and B) and elemental mapping of Ni, Co, C, N, and O (C) for Ni-Gly/ZIF-67. SEM images (D and E) and elemental mapping of Ni, Co, Fe, N, C, and O (F) for Ni-Gly/CoFe-ZIF-67.

The electrochemical performance of the electrode materials was verified through Cyclic Voltammetry (CV) and Electrochemical Impedance Spectroscopy (EIS), and the results are displayed in Figure 3A and 3B, respectively. It is visible that ZIF-67 and CoFe-ZIF-67 electrode materials presented a poor electrochemical behavior when compared to other Ni-Gly-containing electrode materials. In fact, the cyclic voltammogram of the electrode modified with ZIF-67 displayed two pairs of waves related to the Co^2+/3+^ e Co^3+/4+^ redox process^21,23^ located at 0.11/0.10 V and 0.47/0.45 V, respectively, in addition to a low anodic peak current (0.27 and 0.47 mA) associated with those redox processes. For the CoFe-ZIF-67 electrode material, only one pair of waves is visible at 0.18/0.11 V with a current peak of 0.47 mA. The second pair of waves cannot be observed in the cyclic voltammogram because an intense exponential anodic current is observed, due to the oxygen evolution reaction (OER). The poor electrochemical performance of ZIF-67 and CoFe-ZIF-67 is due to their low conductivity and instability.^24,25^ On the other hand, the FTO modified with Ni-Gly exhibited superior electrochemical behavior compared to ZIF-67 and CoFe-ZIF-67. Indeed, the Ni-Gly electrode material exhibited a pair of waves at 0.45/0.28 V, due to the Ni^2+^/^3+^ redox process,^26^ and an intense anodic peak current of 3.9 mA. The well-defined electrochemical behavior of Ni-Gly compared to ZIF-67 and CoFe-ZIF-67 electrode materials can be explained by the *in situ* transformation into Ni­(OH)_2_. It is essential to note that, although a Ni-Gly is obtained using the Tower method,^13^ the Ni­(OH)_2_ is obtained after a few voltammetric cycles (electrochemical activation), as illustrated in Figure S3A.

**Figure 3:**
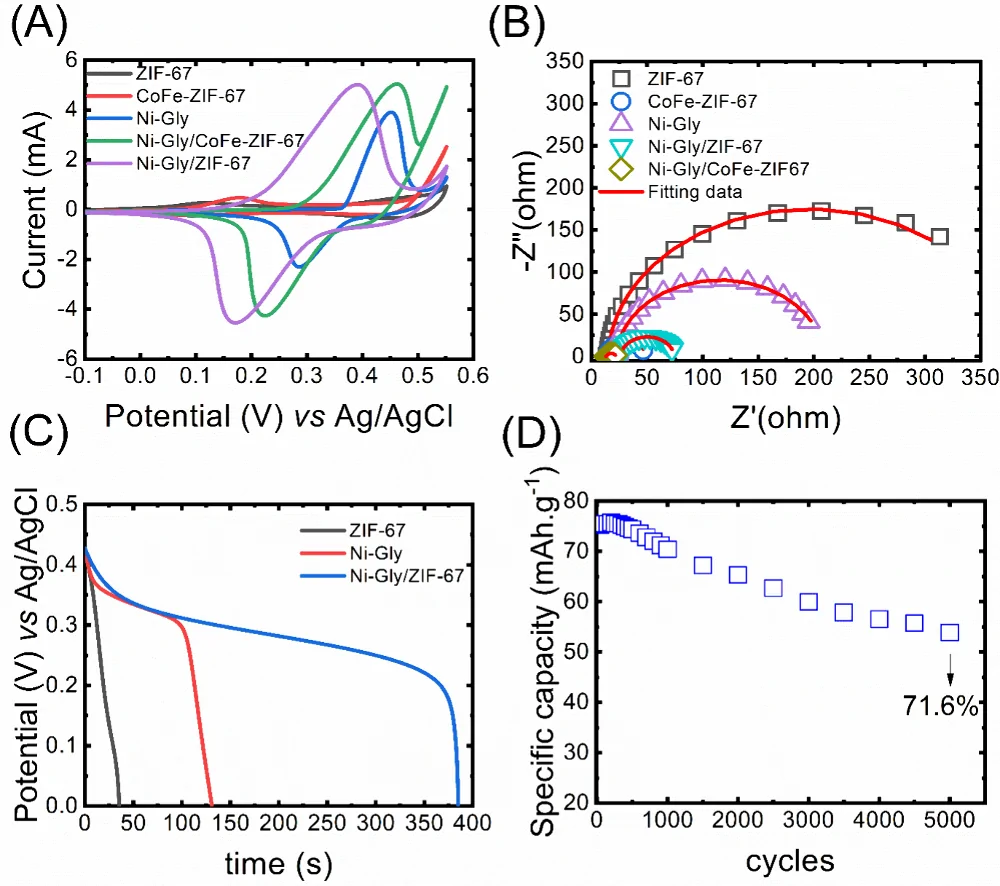
Electrochemical findings. (A) Cyclic voltammograms and (B) Nyquist impedance spectra of FTO electrodes modified with ZIF-67, CoFe-ZIF-67, Ni-Gly, Ni-Gly/ZIF-67, and Ni-Gly/CoFe-ZIF-67. (C) Comparison of potentiometric discharging times for the selected electrodes. (D) Long-term cycling stability (capacity retention) of the Ni-Gly/ZIF-67 electrode at 12 A g^–1^.

The binary electrodes based on Ni-Gly/ZIF-67 and Ni-Gly/CoFe-ZIF-67 presented a single pair of waves at *E*_pa_/*E*_pc_ 0.39/0.17 V (Ni-Gly/ZIF-67) and *E*_pa_/*E*_pc_ 0.46/0.22 V (Ni-Gly/CoFe-ZIF-67). In addition, the binary electrode materials showed an intense anodic current of around 5 mA, and the cyclic voltammograms showed a larger enclosed area than those of the other modified electrodes, suggesting that these binary films have a greater capacity to store energy than pristine materials. It is also worth noting that Ni-Gly/CoFe-ZIF-67 exhibits the same electrochemical behavior for the OER process as CoFe-ZIF-67, as shown in Figure 3A.

In addition to the CV, EIS was performed on the different electrode materials. These findings were presented using the Nyquist plots as shown in Figure 3B and Figure S3. The qualitative analysis of the EIS spectra revealed a high-frequency arc, indicative of the resistive-capacitive behavior of porous materials. At the same time, we verified the medium- to low-frequency semicircle corresponding to the Faradaic process (*Z*_F_ = *R*_ct_), in the absence of mass-transport limitations, which resides in parallel with the electrical double-layer capacitance.

During the fitting/simulation procedure using the CNLS method in ZView software, it was verified the so-called porous-electrode behavior (e.g., φ ≈ −45° at ω → ∞), i.e., the presence of a short linear region at high frequencies with equal imaginary and real parts since φ ≈ −45°), which is coupled with the charge transfer at the pore walls. The latter is a necessary condition for identifying the influence of different pore structures on the impedance response. It is worth noting that the porous electrode behavior manifests in the opposite region of the complex-plane plots, where the so-called Warburg behavior for mass transport is observed (e.g., φ ≈ −45° at ω → 0).

Gassa et al.^27^ and Lasia^28^ discussed the basic theory for studying the impedance behavior of porous/defective electrodes used as electrocatalysts. In the absence of mass-transport limitations, the overall cell impedance (*Z*) is given as follows

8 $$Z=R_{sol}+\frac{R_{pore}}{\Lambda^{1/2}}\coth\left(\Lambda^{1/2}\right)$$

where $\Lambda =\frac{1}{A}+\frac{AB{\left(j\omega \right)}^{\phi }}{A}\approx \frac{1}{A}+B{\left(j\omega \right)}^{\phi }$ , when ϕ ≈ 1 (negligible frequency dispersion).

The limiting cases of eq 8 are (*i*) high frequencies (ω → ∞), where *Z* = *R*_sol_ + *R*_pore_/Λ^1/2^ and φ = – 45°, and (*ii*) low frequencies (ω → 0), where *Z* = *R*_sol_ + *R*_pore_ and φ = 0°.

The different model parameters available from simulations using ZView and other CNLS software are *R*_sol_, *R*_pore_, *A*, *B*, and ϕ. Considering a cylindrical pore geometry, we can obtain the practical parameters describing the electrochemical electrode behavior: σ = *r*/2*ρl* ^2^, *R*_ct_ = *A*/σ, *R*_pore_ = *ρl*/*n*π*r* ^2^, *C*_edl_ = *Bσ*, and *R*_sol_. In this symbology, *A* (−) is a dimensionless simulation parameter, σ (Ω^–1^ cm^–2^) is the areal pore conductance, ρ (Ω cm) is the intrapore electrolyte resistivity, *R*_sol_ (Ω) is the uncompensated solution resistance, *R*_pore_ (Ω) is the cylindrical pore resistance, *R*_ct_ (Ω cm^2^) is the areal charge-transfer resistance, *C*_edl_ (F cm^–2^) is the areal electrical double-layer capacitance, ϕ (−) is the dimensionless constant phase element exponent, *r* (cm) is the average pore radius, *l* (cm) is the average pore length, and *n* (−) is the total number of identical cylindrical pores.

As verified, the Nyquist profiles characterized by a high-frequency short straight line connected to an almost perfect low-frequency semicircle, correspond to the so-called general porous case, where the ac signal penetration length is comparable with the pore length.^28^ We applied unitary pore-dimension and conductance analysis (σ = *r*/2*ρl* = 1) to facilitate data comparison and to verify gains in the electrocatalytic activity of the materials using charge-transfer resistance values. This is a common practice in advanced modeling routines for comparing the electrocatalytic behavior of different materials using EIS.^28^

All simulation findings are presented in Table 1. The CNLS simulations were excellent, with chi-square values below 10^–5^. All relative errors for the individual fitted model parameters were less than 5%.

**Table 1:** Electrochemical Parameters Obtained by Fitting the Porous Electrode Model (eq 8) to the EIS Findings

Electrode Material	*R*_sol_ (Ω cm^2^)	*R*_ct_ = *A*/σ (Ω cm^2^)	*C*_edl_ = *Bσ* (mF cm^–2^)	ϕ (−)	*R*_pore_ (Ω)	R.E.A. (Ω^–1^ cm^–2^)	I.C.A. (mF Ω^–1^ cm^–4^)
**ZIF-67**	13.54	109	67	0.978	3.328	0.00917	0.61
**CoFe-ZIF-67**	13.03	8.78	62	0.961	3.980	0.114	7.1
**Ni-Gly**	20.89	35.36	43	0.985	5.256	0.028	1.2
**Ni-Gly/ZIF-67**	24.67	10.93	252	0.979	4.398	0.091	23
**Ni-Gly/CoFe-ZIF-67**	12.84	4.11	135	0.923	2.055	0.243	33

Areal capacitance (*C*_edl_) values were extracted from CPE (*Z*_cpe_ = 1/*Y*_0_(*jω*)^ϕ^) using the Brug-Sluyters formula:^28^

9 $$C_{edl}={Y_{0}}^{1/\phi }{\left(\frac{1}{R_{sol}}+\frac{1}{R_{ct}}\right)}^{1-1/\phi }$$

The fact that ϕ ≥ 0.923 (see Table 1) confirms a negligible presence of capacitance dispersion phenomena at the pore walls for the different materials.

Symbology: *A* (−) is a dimensionless simulation parameter, σ (Ω^–1^ cm^–2^) is the areal pore conductance, ρ (Ω cm) is the intrapore electrolyte resistivity, *R*_sol_ (Ω) is the uncompensated solution resistance, *R*_pore_ (Ω) is the cylindrical pore resistance, *R*_ct_ (Ω cm^2^) is the areal charge-transfer resistance, *C*_edl_ (F cm^–2^) is the areal electrical double-layer capacitance, ϕ (−) is the dimensionless constant phase element exponent, *r* (cm) is the average pore radius, *l* (cm) is the average pore length, and *n* (−) is the total number of identical cylindrical pores.

The comparative analysis based on Table 1 reveals that *R*_ct_ varies significantly for the investigated materials. Since the relative electrocatalytic activity (R.E.A.) for electrode reactions is proportional to the inverse of the *R*_ct_ values (R.E.A ∝ 1/*R*_ct_), we have the following R.E.A sequence Ni-Gly/CoFe-ZIF-67 > CoFe-ZIF-67 > Ni-Gly ZIF-67 > Ni-Gly > ZIF-67. Therefore, electron transfer is strongly facilitated in the presence of Ni-Gly/CoFe-ZIF-67. On the other hand, electron transfer is significantly hindered in the ZIF-67 system, thereby blocking the water-splitting reaction. The *R*_pore_ values are quite similar (*e.g*., 2.055 to 4.398 Ω), indicating the different materials have a similar degree of porosity. By contrast, the *C*_edl_ value, representing the electrochemically active surface area, changed significantly. These findings indicate the intrinsic electrocatalytic activity (I.C.A = 1/(*R*_ct_/*C*_edl_)), where R.E.A values are normalized by the areal capacitances, is represented by the following sequence Ni-Gly/CoFe-ZIF-67 > Ni-Gly/ZIF-67 > CoFe-ZIF-67 > Ni-Gly > ZIF-67. Therefore, the best overall electrocatalytic performance is obtained for the Ni-Gly/Co–Fe-ZIF-67 material.

Figure S4A-C shows galvanostatic charge–discharge (GCD) curves for ZIF-67, Ni-Gly, and Ni-Gly/ZIF-67 electrode materials in different specific currents (1 – 12 A g^–1^). Through the GCD curve profiles of the compounds, it is possible to classify them as pseudocapacitive or battery-like materials.^28,29^ For instance, it is clear that ZIF-67 exhibits an electrochemical profile distinct from that of Ni-Gly and Ni-Gly/ZIF-67, suggesting that these compounds store energy through different electrochemical processes. The quasi-triangular shape of the GCD curves for ZIF-67 indicates that the material exhibits significant pseudocapacitive properties. In contrast, the GCD curves for Ni-Gly and Ni-Gly/ZIF-67 display low inclined potential plateau, i.e., the potential that remains nearly constant during the redox process is a characteristic feature of battery-like materials.

The inspection of the linear parts of the discharging curves revealed that ZIF-67 has the highest negative slope (−12 mV s^–1^) compared to Ni-Gly (−0.604 mV s^–1^), and Ni-Gly/ZIF-67 (−0.304 mV s^–1^). Thus, we verified that ZIF-67 exhibits high-power characteristics, consistent with a pseudocapacitive charge-storage mechanism, whereas the other materials are more likely to exhibit high-energy characteristics compatible with battery-like charge-storage processes, as shown in Figure S5. It is also possible to observe different discharge times for the electrode materials (Figure 3C), with the value for Ni-Gly/ZIF-67 approximately 3- and 10-fold higher than those for Ni-Gly and ZIF-67, respectively. In fact, the values found for discharge time were 35.6 s (ZIF-67), 131 s (Ni-Gly), and 385.2 s (Ni-Gly/ZIF-67) at 1.0 A g^–1^. By analyzing eq 1, it is clear that the discharge time is directly proportional to specific capacity; therefore, it is expected that the electrode material based on Ni-Gly/ZIF-67 exhibits the highest specific capacity among the electrode materials studied.

According to eq 1, specific capacity can be determined from 1 to 12 A g^–1^; the values are displayed in Figure S4D. Although the ZIF-67 electrode material is more correctly classified as pseudocapacitive, with pseudocapacitance typically expressed in F g^–1^, the specific capacity of the other materials must be expressed in mA h g^–1^ for comparison purposes. Using specific currents of 1 and 12 A g^–1^, the specific capacities obtained were 9.9 and 9.6 mA h g^–1^ (ZIF-67), 36.4 and 26.1 mA h g^–1^ (Ni-Gly), and 106.9 and 93.6 mA h g^–1^ (Ni-Gly/ZIF-67). It can be observed that the capacities of ZIF-67 remained almost unchanged, whereas the capacities of Ni-Gly and Ni-Gly/ZIF-67 decreased as the specific current increased. These results support the presence of distinct energy storage mechanisms in the electrode materials studied here. In fact, the constant specific capacities at different specific currents, with a capacity retention of 96.9%, suggest a fast, reversible surface redox reaction for the ZIF-67 electrode material, consistent with pseudocapacitive behavior. In contrast, Ni-Gly exhibited a capacity retention of 71.7%, indicating that the electrode material performance is strongly dependent on current density, as battery-like materials are governed by solid-state diffusion-controlled Faradaic processes. For Ni-Gly/ZIF-67, an intermediate capacity retention of 87.5% was found, revealing characteristics of both pseudocapacitive and battery-like materials. Given that Ni-Gly/ZIF-67 showed both high specific capacity and excellent capacity retention, this electrode material was selected for further evaluation of its long-term stability. Thus, the binary electrode was subjected to 5,000 consecutive charge/discharge cycles at 12 A g^–1,^ and it retained 71.6% of its capacity, as shown in Figure 3D.

The improved electrochemical performance of Ni-Gly/ZIF-67 may originate from the complementary electrochemical roles of its two components, arising from a structural integration between Ni-Gly and ZIF-67. Ni-Gly provides Faradaic redox-active sites for charge storage, whereas the ZIF-67 framework improves electrolyte accessibility, facilitating ion diffusion and promoting a more homogeneous distribution of electrochemically accessible active sites.

The electrochemical performance of the Ni-Gly/ZIF-67 binary electrode was compared with previously reported materials based on MOF-derived structures and layered double hydroxides (Table 2). Although the specific capacity of Ni-Gly/ZIF-67 (106.9 mA h g^–1^ at 1.0 A g^–1^) is lower than that of some reported systems such as Co-ZIF-67/Ni-CNT@NiLa-LDH (237.5 mA h g^–1^),^30^ V_2_CT_*x*_@NiCoMn–OH-20 (229.8 mA h g^–1^),^31^ ZIF-8/Fe_2_O_3_ (161.2 mAh g^–1^),^32^ and ZIF-67:Ni(1:2) (161.2 mAh g^–1^),^33^ these findings were achieved using highly concentrated electrolytes (e.g., 3–6 M KOH), which are well-known to enhance ionic conductivity and accelerate faradaic reactions. Notably, despite this advantage, the electrode materials based on Co-ZIF-67/Ni-CNT@NiLa-LDH, and V_2_CT_*x*_@NiCoMn–OH-20 exhibited capacity retentions of only 73.3% and 88.44% after 10,000 cycles, respectively, suggesting that highly concentrated electrolytes may also accelerate electrode degradation during prolonged cycling. In contrast, ZIF-8/Fe_2_O_3_ presented a capacity retention of 97%, but the durability test was performed during 1500 cycles of charge/discharge. The Ni-Gly/ZIF-67 electrode developed in this work was evaluated under significantly milder conditions (1.0 M KOH), yet retained 71.6% of its capacity after 5,000 cycles. While this retention value is moderate, it reflects reasonable electrochemical stability under less favorable ionic conditions. Overall, these results suggest that Ni-Gly/ZIF-67 offers a competitive balance between performance and durability, particularly given its practical advantages for operating in dilute electrolytes.

**Table 2:** Electrochemical Performance Comparison of Ni-Gly/ZIF-67 with Selected MOF-Derived and Layered Double Hydroxide Electrodes

Materials	Electrolyte	Specific Capacity	Number of cycles	Retention	ref.
**Co-ZIF-67/Ni-CNT@NiLa-LDH**	KOH (6 M)	237.5 (1.0 A g^–1^)	10000	73%	^30^
**V**_**2**_ **CT**_***X***_ **@NiCoMn-OH-20**	KOH (3 M)	229.8 (1.0 A g^–1^)	10000	88%	^31^
**ZIF-8/Fe**_**2**_ **O**_**3**_	KOH (6 M)	135.3 (2.7 A g^–1^)	1500	97%	^32^
**ZIF-67:Ni**(1:2)	KOH (6 M)	161.2 (1.0 A g^–1^)	5000	74.6%	^33^
**Ni-Mo-MOF**	KOH (1 M)	189.3 (0.5 A g^–1^)	10000	79%	^34^
**Ni**_**10**_ **Fe**_**1**_ **-BDC MOF**	KOH (1 M)	178.6 (12.0 A g^–1^)	2000	72.2%	^35^
**Ni-Gly/ZIF-67**	KOH (1 M)	106.9 (1.0 A g^–1^)	5000	72%	This work

The electrochemical performance of the symmetric supercapacitor (Ni-Gly/ZIF-67//Ni-Gly/ZIF-67) operating in 1.0 M KOH aqueous electrolyte is shown in Figure 4. Although the maximum operating voltage is lower than that of conventional supercapacitors (<1.0 V), the device (Figure 4A) maintains its capacitive behavior in the absence of battery-like redox peaks. This behavior suggests a dual charge-storage mechanism, in which capacitive contributions from the carbonaceous matrix coexist with Faradaic processes associated with the metal-based compounds. The galvanostatic charge–discharge (GCD) curves were obtained within an expanded voltage window up to 0.8 V **(** Figure 4B**)**, defined as the upper potential limit prior to the onset of oxygen evolution. The curves exhibit pseudocapacitive behavior, with quasi-linear, symmetric charge–discharge profiles, indicating efficient charge propagation and a behavior approaching that of an ideal supercapacitor. Also, the device’s rate capability was further evaluated by varying the applied current density (Figure 4C), where the GCD profiles maintained their shape even at higher current densities, suggesting good reversibility with facile transport kinetics. The energy and power densities were evaluated using a Ragone plot (Figure 4D), revealing a maximum energy density of 0.765 W h kg^–1^ at a corresponding power density of 6076 W kg^–1^. Although the energy density remains in the moderate range, the notably high-power density suggests efficient charge transport and low internal resistance within the electrode architecture. These values are competitive with those of similar systems reported in the literature,^36,37^ highlighting the material’s ability to deliver high power while maintaining moderate energy storage. Furthermore, long-term cycling stability was evaluated over 10,000 charge–discharge cycles, and the device retained approximately 79% of its initial capacitance (Figure 4E).

**Figure 4:**
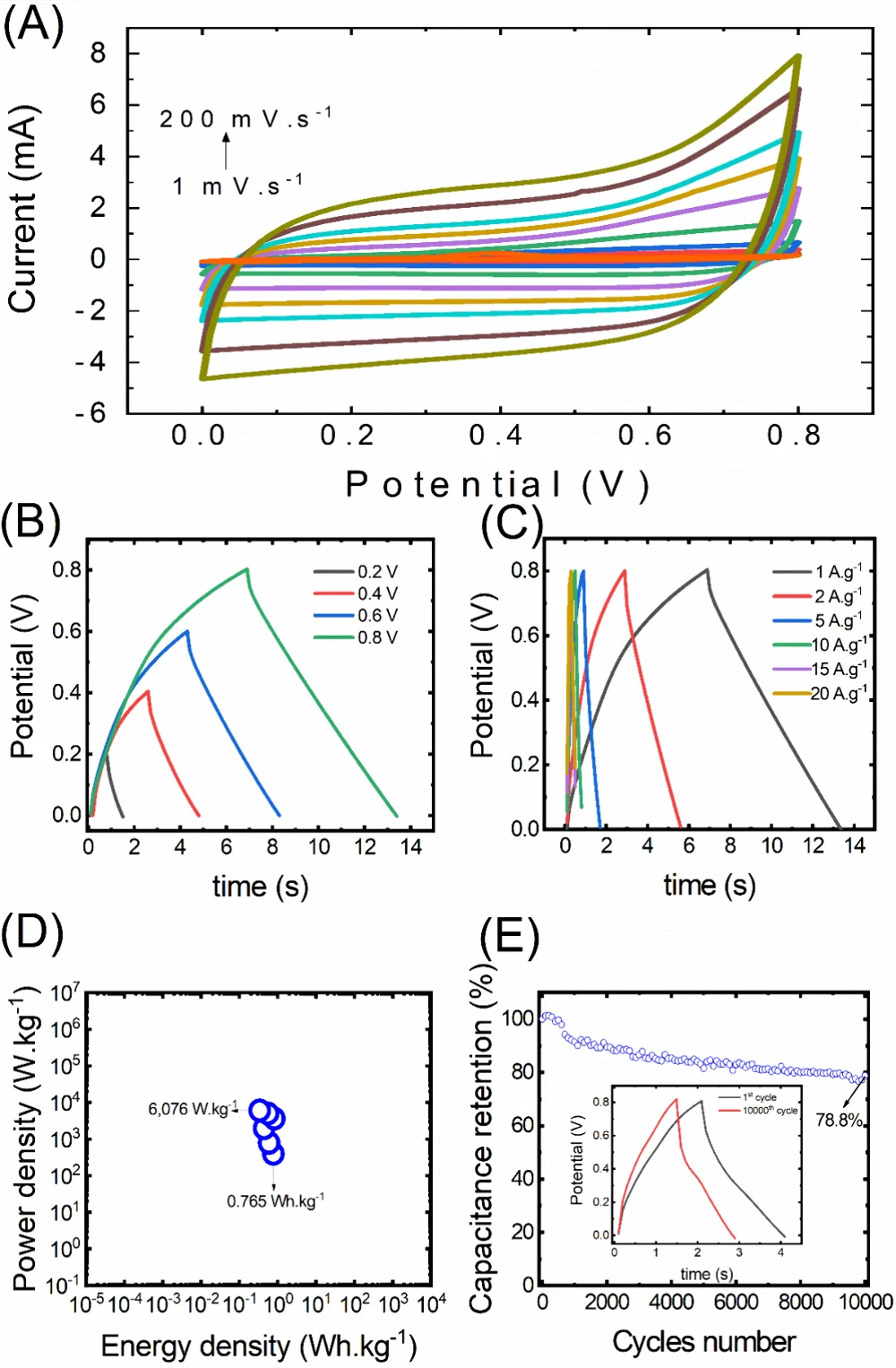
Electrochemical studies of the coin cell electrochemical system (symmetric device) Ni-Gly/ZIF-67//Ni-Gly/ZIF-67 filled with 1.0 M KOH aqueous electrolyte: (A) Cyclic voltammograms with scan rates ranging from 1.0 to 200 mV s^–1^, (B) GCD curves at 1.0 A g^–1^, (C) GCD curves at different current densities, (D) the Ragone plot, (E) Long-term cycling stability. Inset in (E): GCD curves for the 1st cycle and 10,000th cycle.

The catalytic activity of the materials toward the OER was evaluated using LSV. The electrochemical results are presented in Figure 5. As shown in Figure 5A, the overpotential (η) values for the electrodes modified with CoFe-ZIF-67, Ni-Gly, and the Ni-Gly/CoFe-ZIF-67 binary film determined at a current density of 10 mA cm^–2^ were 460 mV, 570 mV, and 430 mV, respectively.

**Figure 5:**
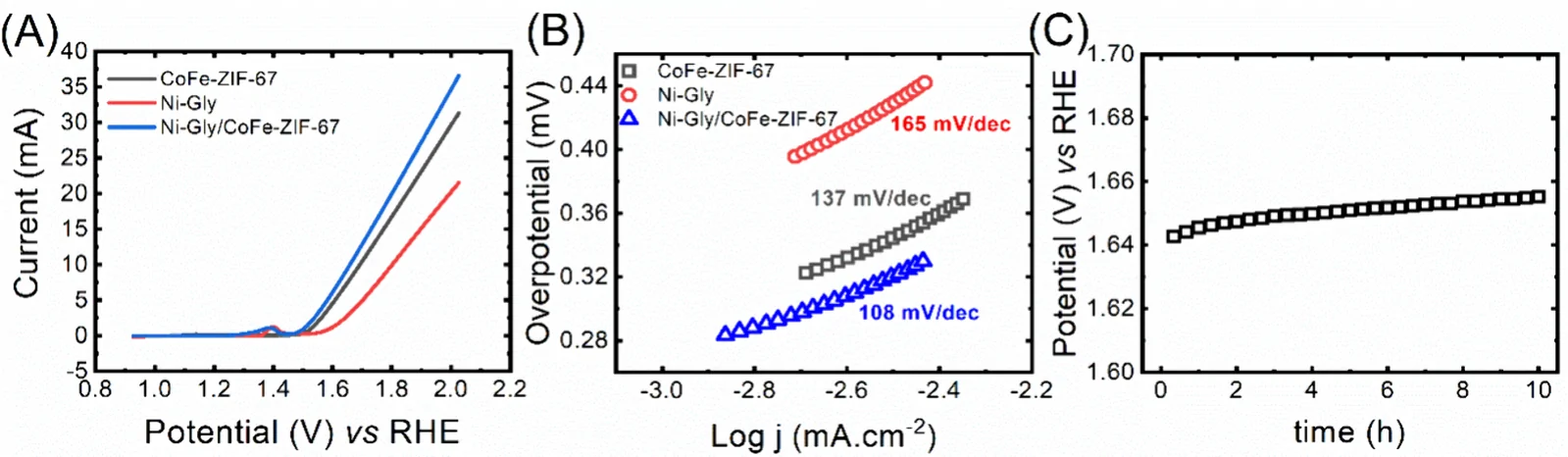
(A) Linear voltammograms for FTO electrodes modified with CoFe-ZIF-67, Ni-Gly, and the Ni-Gly/CoFe-ZIF-67. Scan rate: 5 mV s^–1^. (B) Corresponding Tafel plots derived from the LSV measurements, showing the respective Tafel slope (b) values for each catalyst. (C) Chronopotentiometric curve (stability test) of the Ni-Gly/CoFe-ZIF-67-based electrode at a fixed current density.

Among the investigated materials, the Ni-Gly/CoFe-ZIF-67 demonstrated the lowest overpotential, indicating superior catalytic performance. In addition to these quantitative results from LSV, the observations from CV further corroborated the favorable role of Fe incorporation in facilitating the OER. The introduction of iron ions into the structures, both in CoFe-ZIF-67 and Ni-Gly/CoFe-ZIF-67 electrode materials, clearly enhanced catalytic activity. In fact, according to S. Anantharaj, S. Kundu, and S. Noda^22^ Fe plays several roles in the OER activity of Ni- and Co-based catalysts. In this context, the improved electrocatalytic properties of the binary electrode material can be attributed to the incorporation of Fe, which is expected to contribute through several mechanisms, including: (i) during OER, Ni-Gly turns into catalytically active NiOOH species, and Fe (CoFe-ZIF-67) modifies the electronic structure of NiOOH, adjusting the adsorption energy of the *OH, *O and *OOH intermediates, (ii) increases the electronic conductivity of the metal-oxyhydroxide, although this is not the dominant factor; (iii) promotes structural reorganization, often leading to the formation of Layer Double Hydroxide-type phases; (iv) acts as a strong Lewis acid, polarizing the Ni–O bonds and favoring the formation of highly oxidizing Ni species. Although the exact role of Fe in enhancing OER activity remains the subject of debate in the literature,^22^ it is widely accepted that its incorporation modulates the electronic structure of the catalyst. Indeed, this modulation is evident by XPS analysis, as evidenced by a shift in the binding energies of the Ni 2p peaks in Ni-Gly/CoFe-ZIF-67, suggesting an electron-transfer effect between Ni, Co, and Fe. This contribution was anticipated, given that the OER involves the transfer of four electrons and, consequently, four protons.

The Tafel plots in Figure 5B enabled a deeper analysis of the reaction kinetics. The Tafel slope (*b*) corresponds to the number of millivolts required to increase the current density by 1 order of magnitude (*b*/mV dec^–1^) and is described by the Tafel equation: (η = *a* + *b*log­(*j*)), where *b* is obtained by linear fitting analysis of the overpotential in the linear region (or high-field approximation) of the plot.^38,39^ Lower values of *b* indicate more favorable reaction kinetics, since smaller increases in overpotential are required to achieve a 10-fold increase in current density.^40^ In this study, the Ni-Gly/CoFe-ZIF-67 exhibited the lowest Tafel slope (108 mV dec^–1^) compared to CoFe-ZIF-67 (137 mV dec^–1^) and Ni-Gly (165 mV dec^–1^), confirming its superior catalytic activity. These results are consistent with the linear sweep voltammetry analyses, consolidating the Ni-Gly/CoFe-ZIF-67 binary electrode as the most promising OER catalyst among the investigated materials.

The long-term electrochemical stability of the Ni-Gly/CoFe-ZIF-67 electrode material, which exhibited the best OER performance, was evaluated by chronopotentiometric measurements at 10 mA cm^–2^ for 10 h; the resulting curve is shown in Figure 5C. The electrode demonstrated stable performance, with only a slight potential increase from 1.644 to 1.655 V vs. RHE over the testing period. This minor variation can be attributed to partial leaching of the electroactive material, which requires a small additional potential to maintain the constant current density.^41^

The oxygen evolution reaction (OER) performance of the Ni-Gly/CoFe-ZIF-67 electrocatalyst was compared with that of several electrocatalysts reported in the literature (Table 3).

**Table 3:** Comparison of OER Performance of Ni-Gly/ZIF-67 with Some Electrode Materials

Electrode Material	Electrolyte	η @ 10 mA cm^–2^ (mV)	Tafel Slope (mV dec^–1^)	Stability	ref.
Ni-Mo-MOF	KOH 1 M	290	79	11.1 h	^34^
Ni-Co-ZIF67/MXene	KOH 1 M	270	57.7	16 h	^42^
CoFeZn-Gly	KOH 1 M	335	37.2	24 h	^11^
Mn-Co BTC (1:2)	KOH 1 M	359	71	16 h	^43^
**Ni-Gly/CoFe-ZIF-67**	KOH 1 M	**430**	**108**	**10 h**	**This work**

For comparison, Ni–Mo-MOF demonstrates a lower overpotential of 290 mV with a Tafel slope of 79 mV dec^–1^, although its operational stability is limited to 11.1 h.^34^ Similarly, Ni–Co-ZIF67/MXene achieves improved activity, with an overpotential of 270 mV and a Tafel slope of 57.7 mV dec^–1^, maintaining stability for 16 h.^42^ These enhancements are commonly attributed to the incorporation of highly conductive supports, which facilitate charge transfer and improve the accessibility of active sites. It is important to note that direct comparisons of overpotential and Tafel slope values should consider variations in experimental conditions, including electrolyte composition, catalyst loading, and substrate materials. For instance, the incorporation of conductive materials such as MXene^42^ can enhance charge transfer and increase the accessibility of active sites. Moreover, MXene can contribute to the catalytic behavior through its conductive nature and interfacial interactions with the metal–organic framework. In this context, the Ni-Gly/CoFe-ZIF-67 electrocatalyst demonstrates consistent, reproducible OER activity and stability for 10 h under the tested conditions. Although its overpotential and Tafel slope are higher than those of some optimized systems, the results highlight the viability of this material as a functional OER electrocatalyst without the need for complex hybridization strategies.

## 4 Conclusions

In summary, this study demonstrates the dual electrochemical functionality of the Ni-Gly/MOF binary materials, integrating energy storage and water-splitting applications on a single material platform. The synthesis of Ni-Gly/ZIF-67 and Ni-Gly/CoFe-ZIF-67 coordination compounds yielded homogeneous, porous, and multimetallic structures with enhanced charge transport and redox activity. The functional properties were successfully tailored by compositional tuning. Thus, for energy storage applications, the Ni-Gly/ZIF-67 binary electrode exhibited a high specific capacity and excellent cycling stability, supporting its characteristics as a battery-type electrode for charge storage in high-energy devices. In contrast, Fe incorporation into the MOF framework (Ni-Gly/CoFe-ZIF-67) modulated the electronic environment around Ni sites, facilitating the *in situ* formation of active NiOOH species. This significantly enhanced OER activity, resulting in low overpotential, favorable kinetics, and long-term durability. These findings highlight how coupling MOF-derived architectures with Ni-Gly overcomes the intrinsic limitations of each component, such as poor activity and limited stability, thereby enabling multifunctional performance. Overall, this work presents a rational, cost-effective approach to designing sustainable electrode materials that efficiently bridge energy storage and conversion in integrated energy systems.

## Supplementary Material



## Notes

High-resolution XPS spectra of Ni-Gly/CoFe-ZIF-67; SEM images of ZIF-67, CoFe-ZIF-67, and Ni-Gly; electrochemical findings including cyclic voltammograms and high-frequency Nyquist plots; galvanostatic charge–discharge curves and specific capacity evaluation for ZIF-67, Ni-Gly, and Ni-Gly/ZIF-67; linear fitting of the plateau regions in the discharge curves (PDF)
