# Supplementary material for: Nickel-Glycerolate/MOF-Based Electrode Materials for Energy Storage and Water-Splitting Applications
Source: ACS Omega. 2026 Sep 23;11(39):59762–72. doi: 10.1021/acsomega.6c06663 (PMC13648070; doi:10.1021/acsomega.6c06663)
Supplement: Supplementary file 1 [file ao6c06663_si_001.pdf]

Nickel-Glycerolate/MOF-based Electrode Materials for Energy Storage and Water-Splitting Applications

Iara Schlag Durães Campos[a], Abelardo Gregório Banze[a], Tarso Leandro Bastos[a,b], Raylander Rodrigues Silva[a], Niélcio Batista da Silva Filho[c], Leonardo Morais Da Silva[c], Josué Martins Gonçalves[d,e] and Paulo Roberto Martins[a,b*]

[a]*Chemistry Institute, Federal University of Goias, Av. Esperança S/N, Goiânia, GO 74690-900, Brazil*

[b]*Center of Excellence in Hydrogen and Sustainable Energy Technologies. Chemistry Institute, Federal University of Goias, Av. Esperança S/N, Goiânia, GO 74690-900, Brazil*

[c]*Department of Chemistry, Laboratory of Fundamental and Applied Electrochemistry, Federal University of Jequitinhonha and Mucuri's Valley, Rodovia MGT 367, Km 583, 5000, Alto da Jacuba, Diamantina, MG 39100-000, Brazil*

[d]*Mackenzie Institute for Research in Graphene and Nanotechnologies (MackGraphe), Mackenzie Presbyterian Institute, Consolação Street 930, São Paulo, São Paulo 01302-907, Brazil*

[e]*Mackenzie School of Engineering, Mackenzie Presbyterian University, Consolação Street 930, São Paulo, São Paulo 01302-907, Brazil*

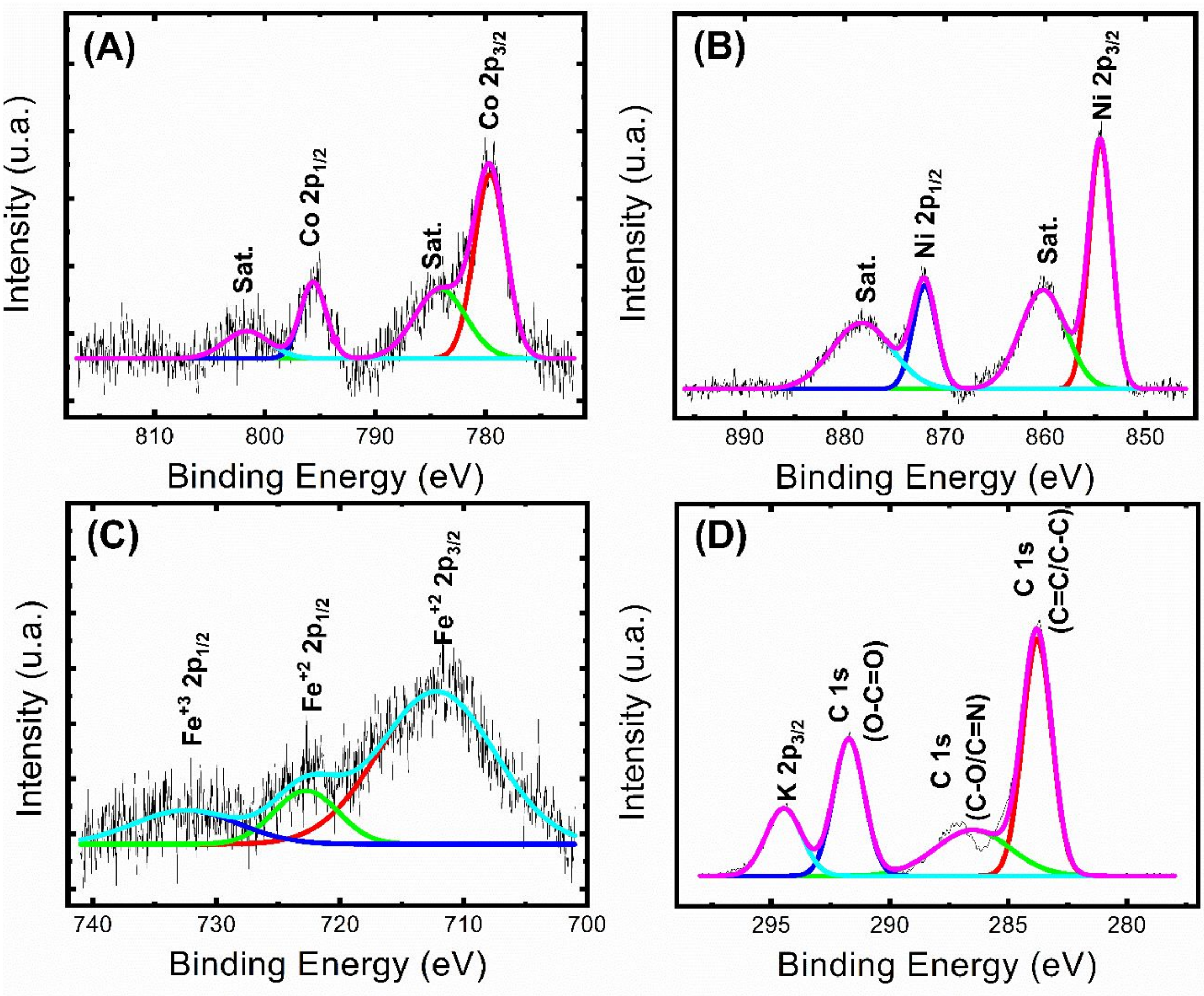

Figure S1: High-resolution XPS spectra of Ni-Gly/CoFe-ZIF-67 for (A) Co 2p, (B) Ni 2p, (C) Fe 2p, and (D) C 1s.

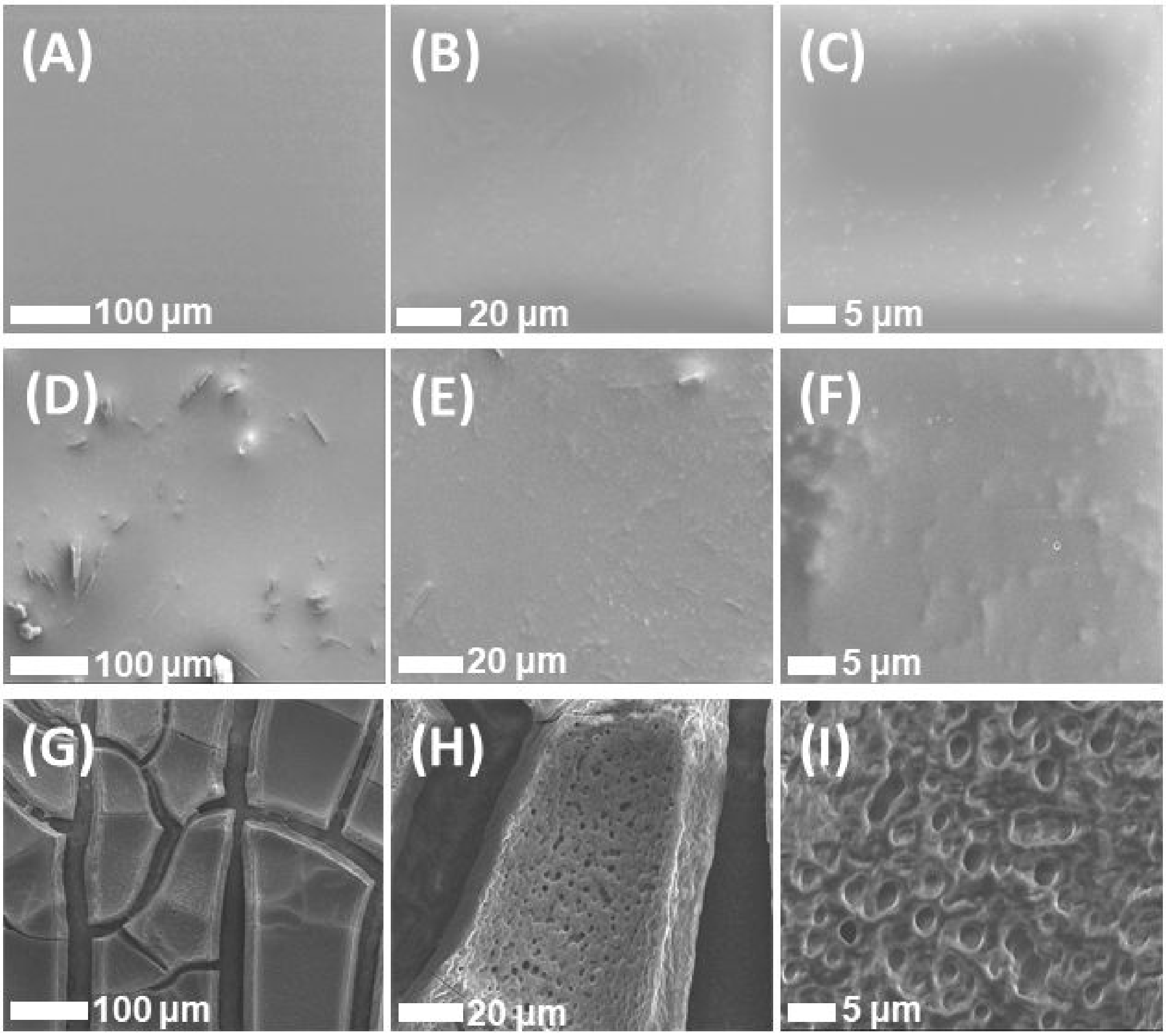

Figure S2: SEM images of (A-C) ZIF-67, (D-F) CoFe-ZIF-67, and (G-I) Ni-Gly.

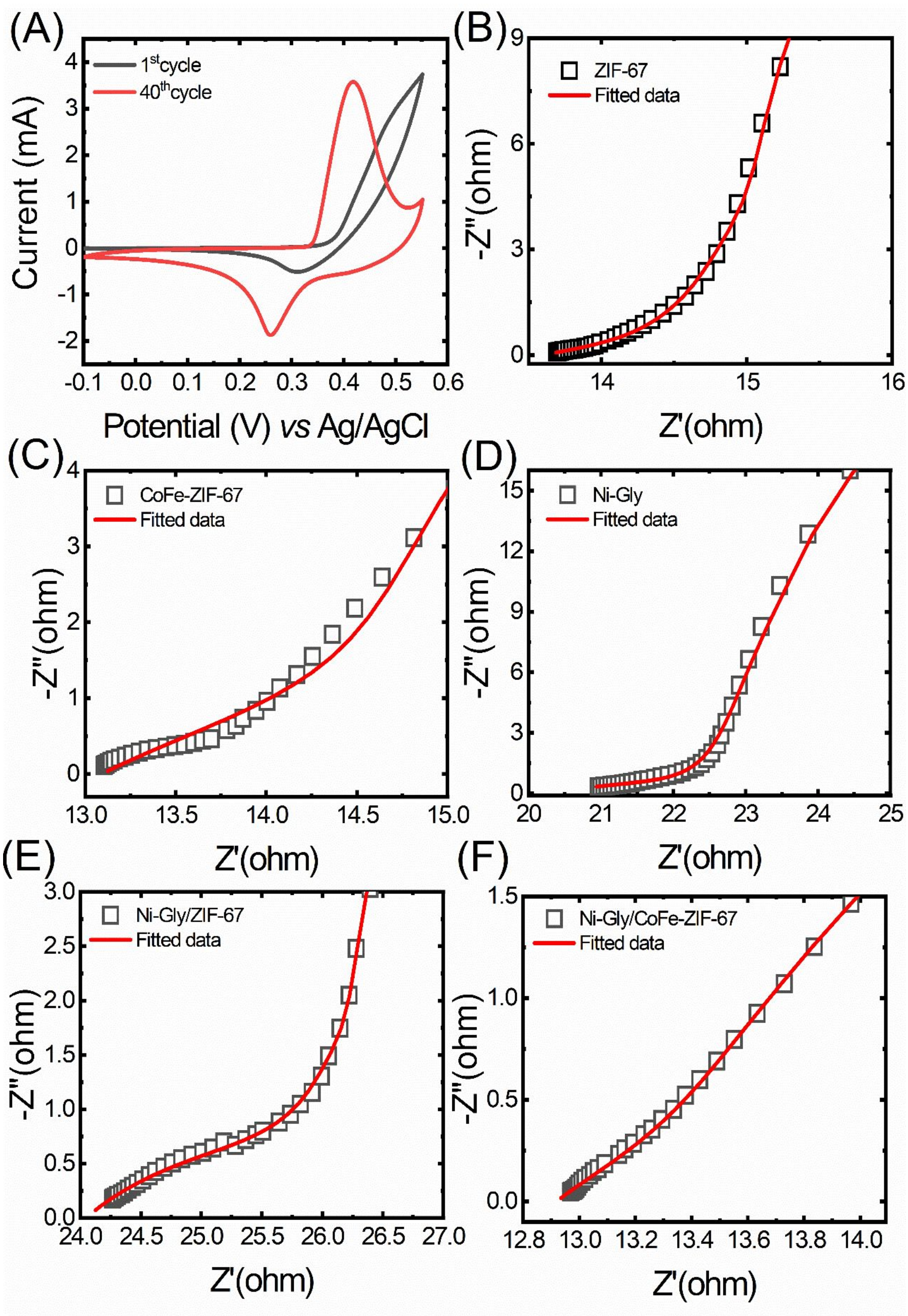

Figure S3: Electrochemical findings. (A) Cyclic voltammograms of the Ni-Gly sample from the 1st to the 40th cycle in 1.0 mol L^{-1} of KOH solution. (B-F) High-frequency Nyquist plots regions.

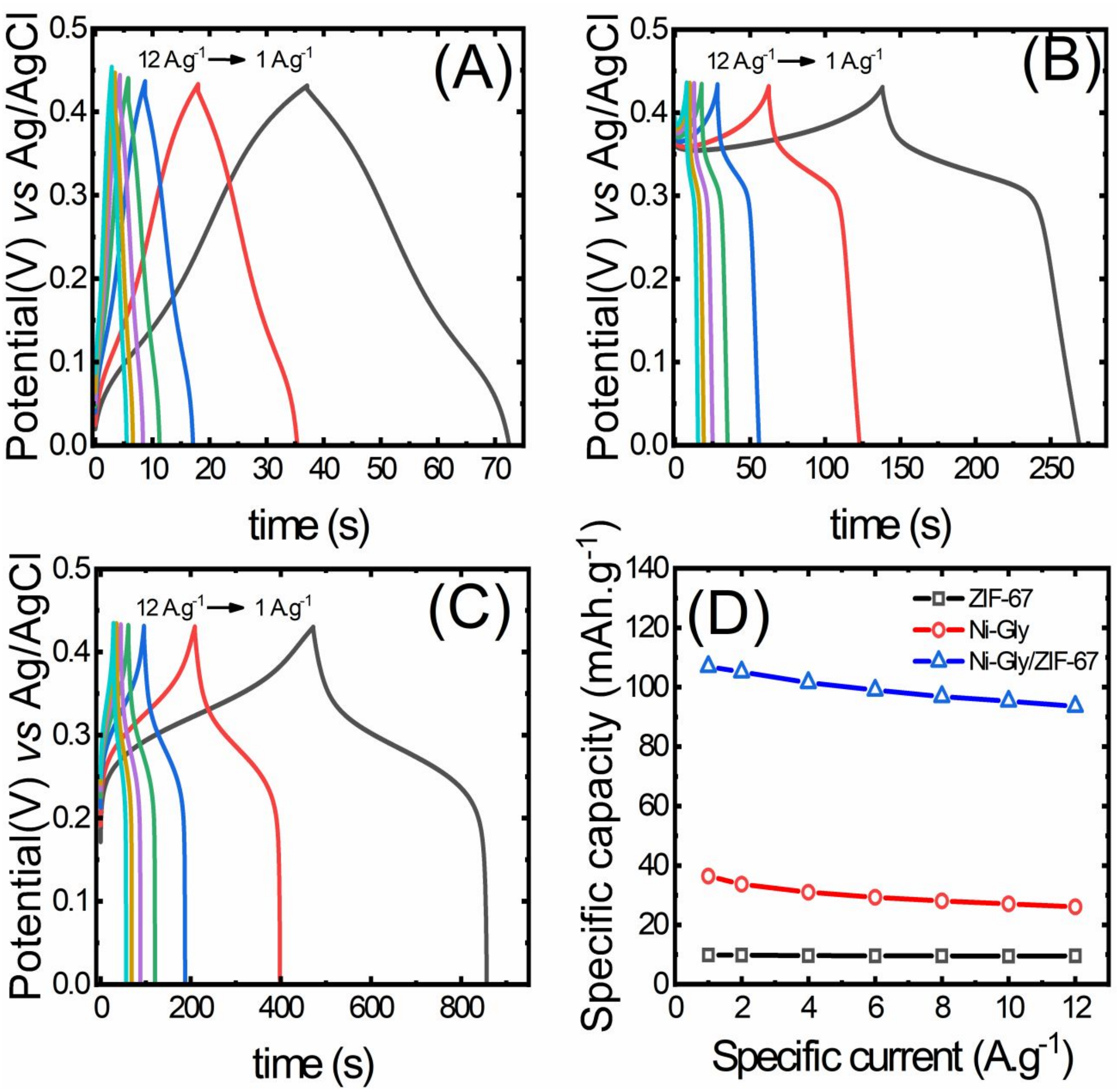

Figure S4: Galvanostatic charge-discharge curves (1 to 12 A g^{-1}) for (A) ZIF-67, (B) Ni-Gly, and (C) Ni-Gly/ZIF-67 electrode materials. (D) Specific capacity at different specific currents for the electrode materials.

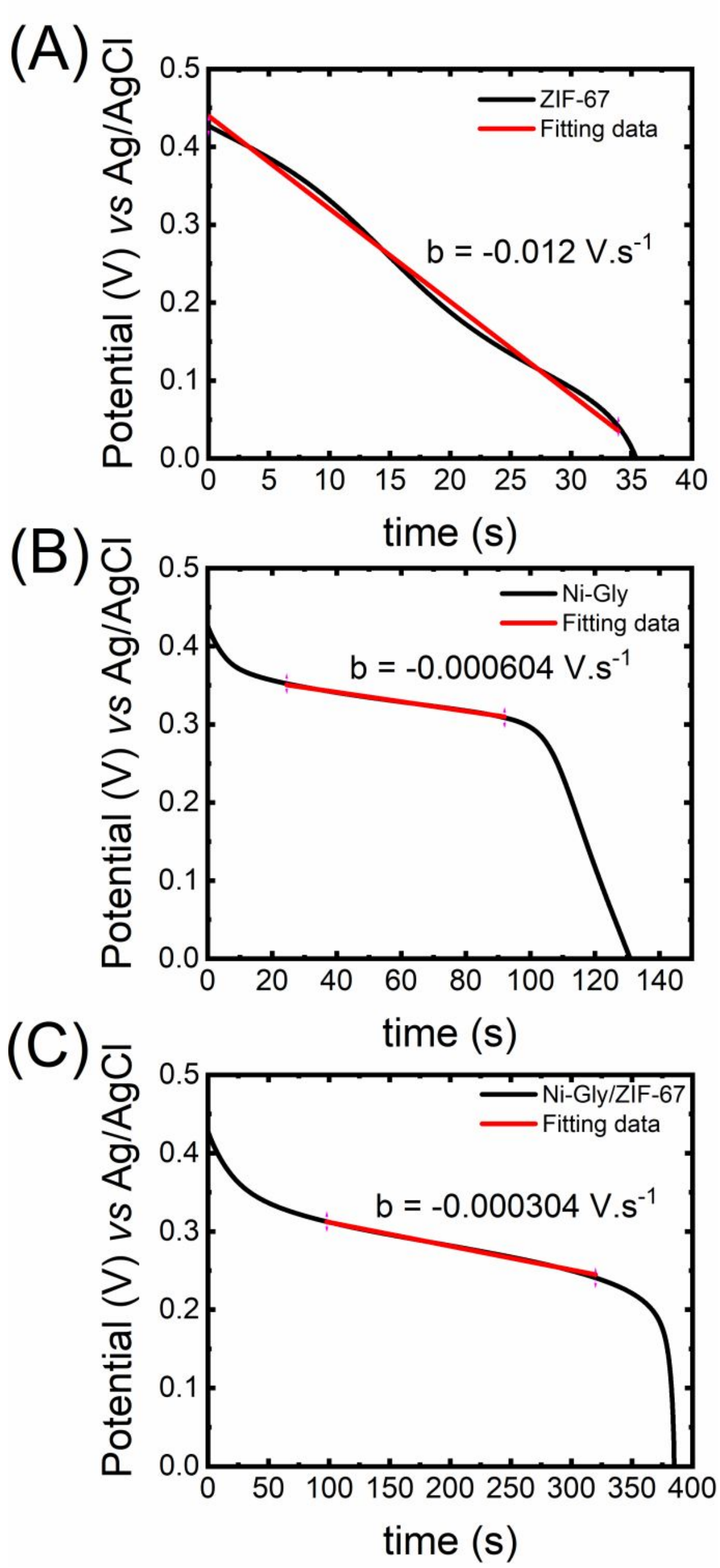

Figure S5: Linear fitting of the plateau regions in the galvanostatic curves of (A) ZIF-67, (B) Ni-Gly, and (C) Ni-Gly/ZIF-67.
